# The impact of age and comorbidity on the postoperative outcomes after emergency surgical management of complicated intra-abdominal infections

**DOI:** 10.1038/s41598-020-58453-1

**Published:** 2020-01-31

**Authors:** Carmen Payá-Llorente, Elías Martínez-López, Juan Carlos Sebastián-Tomás, Sandra Santarrufina-Martínez, Nicola de’Angelis, Aleix Martínez-Pérez

**Affiliations:** 1 Department of General and Digestive Surgery, Hospital Universitario Doctor Peset, Valencia, Spain; 2 Department of Digestive, Hepato-Pancreato-Biliary Surgery, and Liver Transplantation, Henri-Mondor University Hospital, Créteil, France

**Keywords:** Gastrointestinal diseases, Geriatrics, Risk factors, Comorbidities

## Abstract

Age-adjusted Charlson Comorbidity Index (a-CCI) score has been used to weight comorbid conditions in predicting adverse outcomes. A retrospective cohort study on adult patients diagnosed with complicated intra-abdominal infections (cIAI) requiring emergency surgery was conducted in order to elucidate the role of age and comorbidity in this scenario. Two main outcomes were evaluated: 90-day severe postoperative complications (grade ≥ 3 of Dindo-Clavien Classification), and 90-day all-cause mortality. 358 patients were analyzed. a-CCI score for each patient was calculated and then divided in two comorbid categories whether they were ≤ or > to percentile 75 ( = 4): Grade-A (0–4) and Grade-B ( ≥ 5). Univariate and multivariate regression analyses were performed, and the predictive validity of the models was evaluated by the area under the receiver operating characteristics (AUROC) curve. Independent predictors of 90-day severe postoperative complications were Charlson Grade-B (Odds Ratio [OR] = 3.49, 95% confidence interval [95%CI]: 1.86–6.52; p < 0.0001), healthcare-related infections (OR = 7.84, 95%CI: 3.99–15.39; p < 0.0001), diffuse peritonitis (OR = 2.64, 95%CI: 1.45–4.80; p < 0.01), and delay of surgery > 24 hours (OR = 2.28, 95%CI: 1.18–4.68; p < 0.02). The AUROC was 0.815 (95%CI: 0.758–0.872). Independent predictors of 90-day mortality were Charlson Grade-B (OR = 8.30, 95%CI: 3.58–19.21; p < 0.0001), healthcare-related infections (OR = 6.38, 95%CI: 2.72–14.95; p < 0.0001), sepsis status (OR = 3.98, 95%CI: 1.04–15.21; p < 0.04) and diffuse peritonitis (OR = 3.06, 95%CI: 1.29–7.27; p < 0.01). The AUROC for mortality was 0.887 (95%CI: 0.83–0.93). Post-hoc sensitivity analyses confirmed that the degree of comorbidity, estimated by using an age-adjusted score, has a critical impact on the postoperative course following emergency surgery for cIAI. Early assessment and management of patient’s comorbidity is mandatory at emergency setting.

## Introduction

Complicated intra-abdominal infections (cIAI) are the second most common site of invasive infections in critically ill patients^1^. They are associated with poor outcomes in high risk patients, with an estimated overall mortality ranging from 10% to 35%^2–4^. cIAI implies the extension of the process beyond the organ to the peritoneal cavity and is then associated with localized or diffuse peritonitis. A landmark multi-centric international prospective cohort study, evaluated adult patients presenting with cIAI undergoing surgery or interventional drainage and identified the independent risk factors of mortality^3^. They were namely patient’s age, immunosuppression, small bowel perforations, a delay of initial intervention over 24 hours, and intensive care unit (ICU) admission. Previous studies on IAI also showed other factors that potentially influence patient’s prognosis, such as an extended peritonitis, sepsis development, or healthcare-related infections^4^. An emergency surgical procedure is often needed in the management of cIAI, leading to a non-despicable cost burden to healthcare systems^5^.

In 1987, Charlson and colleagues^6^ proposed a new method for classifying the degree of comorbidities in longitudinal studies in order to estimate the probability of death, the Charlson Comorbidity Index (CCI). In 1994, the same group modified the index, taking into account the influence of patient’s age, then creating the age-adjusted CCI (a-CCI)^7^. The score includes 19 medical conditions weighted between 1 to 6 points. Additionally, 1 point is aggregated for every decade after 40 years of age. The score has been widely used at studies evaluating surgical and non-surgical scenarios to weight comorbid conditions in predicting adverse outcomes^8,9^. However, no previous study evaluated the score, nor the influence of the number and degree of comorbidities, in the setting of cIAI.

Therefore, the aim of the present study was to elucidate the role of the patient’s comorbidity adjusted by age^7^, within other potential risk factors of postoperative adverse events, on the outcomes after emergency surgical procedures for cIAI treatment.

## Methods

The present study is a part of a retrospective evaluation involving all the patients with suspected or confirmed COMplicated INtra-abdOminal infections, the *COMINO Project*, admitted at the Department of General and Digestive Surgery from Doctor Peset University Hospital (Valencia, Spain) from January 2014 to December 2017. The present study was performed in accordance to the latest version of the Declaration of Helsinki. The study protocol was reviewed and approved by the local ethics committee (CEIm74/19). Written informed consent of the retrospectively included patients was waived according to local legislation. From the original database, data from adult patients ( > 18 years old) diagnosed with cIAI during emergency surgery were extracted and further analyzed. Patients with suspected cIAI receiving other treatments than surgery, or presenting with post-traumatic, gynecologic or urinary sources were not included in the analyses.

Two main outcomes were evaluated: 1) 90-day severe postoperative complications, defined as grade ≥ 3 of the Dindo-Clavien Classification;^10^ and 2) 90-day all-cause mortality. Data was extracted from electronic clinical report forms. Variables being part of the a-CCI score were extracted by two independent researchers, and all disagreements between them were resolved by discussion with a third one. For the assessment of comorbidity, the total a-CCI score^7^ for each patient was calculated and further dichotomized in two comorbid categories by the percentile 75 ^11^: Grade A ( ≤ percentile 75), Grade B ( > percentile 75). A number of other variables being considered potentially associated with cIAI adverse outcomes after surgery were evaluated, including: sex, obesity (Body Mass Index [BMI] > 30 kg/m^2^), healthcare-associated infections (developed in hospitalized patients or residents of long term healthcare facilities)^12^, diffuse peritonitis, source of infection, sepsis status (defined according to the American College of Chest Physicians/Society of Critical Care Medicine [ACCP/SCCM] Consensus Conference^13^) and delayed initial intervention ( > 24 h) from admittance at the emergency department.

### Statistical analysis

Descriptive data is expressed as mean (standard deviation [SD]) or median (inter-quartile range [IQR]), and n (%) as appropriate. The predictive factors of 90-day severe postoperative complications and 90-day all-cause mortality were assessed using univariate and multivariate analyses. Pearson’s Chi-Square or Fisher’s Exact tests were employed as appropriate. Multivariate stepwise logistic regression analyses were used to adjust for multiple predictive factors and their interactions. The 0.1 level was defined for entry into the model. Multivariable *x*^2^ and *p* values were used to characterize the independence of these factors. Odds ratio (OR) and 95% confidence interval (95%CI) were used to quantify the relationship between the outcomes of interest and each independent factor. All the tests were 2-sided, and the threshold of significance was set at p < 0.05. Multivariate goodness-of-fit was tested using Hosmer-Lemeshow test, and model performance with Nagelkerke *R*^2^. The predictive validity of the models was assessed by calculating the area under the receiver operating characteristics (AUROC) curve. The accuracy determined by the AUROC curve was interpreted as poor if within 0.51 and 0.69; useful if within 0.70 and 0.79; and good if ≥ 0.80^14–16^. Statistical analyses were performed using Statistical Package for Social Sciences software (Statistical Package for Social Science, IBM SPSS Statistics, Version 24 for Macintosh; IBM Corp., Armonk, NY, USA). The results were reported according to the strengthening the reporting of observational studies in epidemiology (STROBE) statement guidelines^17^.

## Results

**Figure 1:**
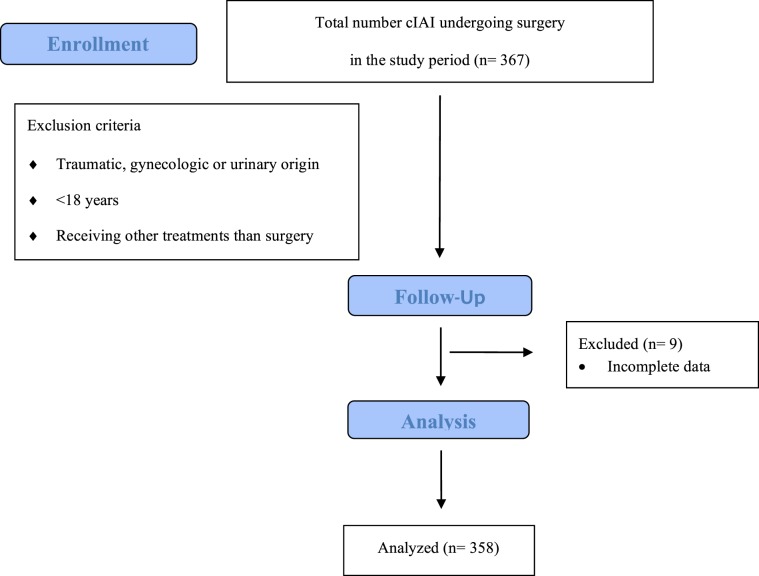
Patient flow-chart.

Out of 571 records of patients presenting with suspected or confirmed cIAI in the study period, 367 (64%) patients diagnosed with cIAI at surgery and fulfilling the inclusion criteria were selected. Nine patients were excluded from the analysis due to missing data (Fig. 1). Thus, 358 patients with a mean age of 58.2 years (SD 19.2) were analyzed, 202 of them (56.4%) being men. The a-CCI median score was 2 (IQR 0–4). Dichotomization of the a-CCI was then established as follows: Grade-A (0–4) and Grade-B ( ≥ 5). Main comorbidities found were diabetes (11.7%), chronic pulmonary disease (8.7%), and coronary disease (8.4%) (Table 1). The distribution of comorbid categories according to the a-CCI score was: Grade A 277 (77.4%), Grade B 81 (22.6%). Fifty-five patients (15.4%) presented with healthcare-associated infections. Complicated appendicitis was the most common origin (46.1%), followed by colorectal (16.4%) and postoperative sources (11.5%). Demographic, clinical, and diagnostic features are displayed in Table 2.

**Table 1:** Frequency of Charlson Comorbidity Index Conditions (n = 358).

Index Weight	Condition	Frequency % (n)
1	Coronary artery disease^a^	8.4 (30)
1	Congestive heart failure	3.4 (12)
1	Peripheral vascular disease	1.1 (4)
1	Cerebrovascular disease	1.4 (5)
1	Dementia	0.8 (3)
1	Chronic pulmonary disease	8.7 (31)
1	Connective tissue disease	0.6 (2)
1	Ulcer disease	2 (7)
1	Mild liver disease	3.4 (12)
1	Diabetes	11.7 (42)
2	Hemiplegia	0 (0)
2	Moderate or severe renal disease	3.6 (13)
2	Diabetes with end-organ damage	1.4 (5)
2	Any tumor^b^	6.9 (25)
2	Leukemia	0.6 (2)
2	Lymphoma	0
3	Moderate or severe liver disease	1.7 (6)
6	Metastatic solid tumor	3.4 (12)
6	AIDS	0

^a^Including myocardial infarction, coronary artery bypass graft, percutaneous transluminal coronary angioplasty and angina pectoris.

^b^Except basal cell skin carcinoma.

*Each decade of age* ≥ *40 years is equivalent to a 1-point increase in comorbidity (i.e., 50–59 years* = *1 point; 60–69 years* = *2 points). Charlson M. J Clin Epidemiol. 1994; 47(11): 1245-51*.

**Table 2:** Demographics, clinical presentation and diagnosis (n = 358).

Age (yr) [mean (SD)]	58.2 (±19.2)
Male Gender [n (%)]	202 (56.4)
BMI (kg/m^**2**^) [mean (SD)]	26.9 (±5.8)
Obesity (BMI ≥ 30 Kg/m2) [n (%)]	57 (15.9)
a-CCI score [median (IQR)]	2 (0–4)
Grade A [0–4] [n (%)]	277 (77.4)
Grade B [ ≥ 5] [n (%)]	81 (22.6)
**Symptoms and signs at admittance**	
Abdominal Pain [n (%)]	345 (96.4)
Abdominal tenderness [n (%)]	290 (81)
Fever ( > 38 °C) [n (%)]	106 (29.6)
**Biomarkers**	
Neutrophil count [median (IQR)]	11700 (8200–15200)
Leucocyte count [median (IQR)]	13700 (10275–17500)
C- Reactive Protein level [median (IQR)]	112 (31.4–223)
Hemoglobin level (g/L) [mean (SD)]	13.2 (±2.3)
**Sepsis** [n (%)]	
No sepsis	100 (27.9)
Sepsis	258 (72)
**Healthcare-related infections** [n (%)]	55 (15.4)
**Radiological assessment** [n (%)]	
Ultrasound	219 (61.2)
CT-Scan	175 (48.9)
**Source of CIA** [n (%)]	
Appendicitis	165 (46.1)
Cholecystitis	30 (8.4)
Colorectal	59 (16.4)
Gastro-duodenal perforation	37 (10.3)
Small bowel perforation	21 (5.9)
Post-operative	41(11.5)
Other	5 (1.4)

Abbreviations: *BMI* stands for body mass index*; ASA* for American Society of Anesthesiolog*y; CT-Scan* for Computer tomography scan.

The median delay of surgery was 7 hours (IQR 4–14). In 52 patients (14.8%), the delay was longer than 24 hours. Laparoscopic treatment was performed in 65.6% of the patients and the overall conversion rate was 5%. Diffuse peritonitis was found in 144 patients (40.2%). The median duration of hospital stay was 7 days (IQR 5–7). 90-day postoperative complications were appeared in 157 patients (43.9%), with severe complications (Dindo-Clavien grades ≥ 3) occurring in 75 (20.9%) of them. All-cause 90-day mortality rate was 9.8%. Surgical and postoperative variables are displayed in Table 3.

**Table 3:** Surgical and postoperative outcomes.

**Delay of surgery after admittance**	
Time (hours) [median (IQR)]	7 (4–14)
Delay ≥ 24 h [n (%)]	52 (14.8)
**Laparoscopic treatment** [n (%)]	235 (65.6)
Conversion to laparotomy [n (%)]	18 (5)
**Degree of peritonitis** [n (%)]	
Focal	214 (59.8)
Diffuse	144 (40.2)
**Operative time (min)** [median (IQR)]	80 (60–110)
**90-day postoperative complication** [n (%)]	157 (43.9)
Dindo-Clavien [n (%)]	
I	30 (8.4)
II	52 (14.5)
IIIa	18 (5)
IIIb	7 (2)
IVa	12 (3.4)
IVb	3 (1)
V	35 (9.8)
Dindo-Clavien ≥ III	75 (20.9)
**Intensive Care Unit admission**	
Patients [n (%)]	97 (27.1)
Stay [median (IQR)]	4 (2–10)
**Hospital stay, days** [median (IQR)]	7 (5–7)
**90-day mortality** [n (%)]	35 (9.8)

**Figure 2:**
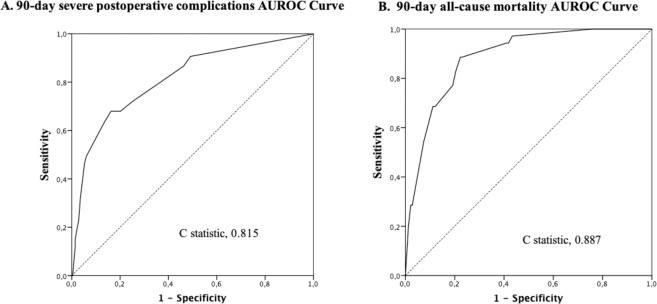
Area Under the Receiver Operating Characteristic (AUROC) in 90-day severe postoperative complications (**A**) and 90-day all-cause mortality (**B**) models. (**A**) AUROC in 90-day severe postoperative complication model 0.815 (95% CI 0.758–0.872). (**B**) AUROC in 90-day all-cause mortality model 0.887 (95% CI 0.83–0.93).

Univariate analyses identified all variables with a potential independent correlation with postoperative adverse outcomes (Table 4). After the multivariate analysis, four variables were found to be independent predictors of 90-day severe postoperative complications: Charlson Grade B (OR = 3.49, 95%CI: 1.86–6.52; p < 0.0001), healthcare-related infections (OR = 7.84, 95%CI: 3.99–15.39; p < 0.0001), diffuse peritonitis (OR = 2.64, 95%CI: 1.45–4.80; p < 0.0001), and delay of surgery more than 24 hours (OR = 2.28, 95%CI: 1.18–4.68; p < 0.024) (Table 4). The model built predicted 90% of 90-day severe postoperative complications in patients presenting all four variables. Hosmer-Lemeshow goodness-of-fit test significance was 0.31. The model correctly classified 84.6% of cases and its performance was tested using Nagelkerke *R*^2^ with a result of 0.36. The AUROC was 0.815 (95%CI: 0.758–0.872) (Fig. 2A).

**Table 4:** Uni- and multivariate analyses on the association between the variables with 90-day postoperative severe complications and 90-day mortality.

Variables	90-day postoperative morbidity					90-day all-cause postoperative mortality				
	Univariate Analysis			Multivariate Analysis		Univariate Analysis			Multivariate Analysis	
	n (%)	Odds Ratio (95%CI)	P value	Odds Ratio (95%CI)	P value	n (%)	Odds Ratio (95%CI)	P value	Odds Ratio (95%CI)	P value
**Sex**										
M	44 (21.8)	1.12	0.66			21 (10.4)	1.17	0.65		
F	31 (19.9)	(0.67–1.88)				14 (9)	(0.57–2.39)			
**BMI (kg/m**^**2**^**)**										
≥30	16 (28.1)	1.33	0.47			9 (15.8)	1.94	0.14		
<30	36 (22.6)	(0.67–2.65)				14 (8.8)	(0.79–4.77)			
**Charlson**										
Grade B (≥5)	36 (44.4)	4.82	0.00*	3.49	0.00*	24 (29.6)	10.18	0.00*	8.30	0.00*
Grade A (0–4)	39 (14.1)	(2.80–8.49)		(1.86–6.52)		11 (4)	(4.72–21.96)		(3.58–19.21)	
**Healthcare-related infections**										
Yes	33 (60)	9.32	0.00*	7.84	0.00*	18 (32.7)	8.18	0.00*	6.38	0.00*
No	42 (13.9)	(4.96–17.50)		(3.99–15.39)		17 (5.6)	(3.88–17.25)		(2.72–14.95)	
**Sepsis**										
Yes	62 (24)	2.17	0.02*	1.98	0.08	32 (12.4)	4.57	0.00*	3.98	0.04*
No	13 (13)	(1.10–4.05)		(0.92–4.28)		3 (3)	(1.36–15.30)		(1.04–15.21)	
**Delay of surgery after admittance**										
≥24hours	20 (37.7)	2.75	0.00*	2.28	0.02*	7 (13.2)	1.5	0.36		
<24hours	55 (18)	(1.47–5.15)		(1.18–4.68)		28 (9.2)	(0.62–3.64)			
**Degree of peritonitis**										
Diffuse	47 (32.6)	3.21	0.00*	2.64	0.00*	25 (17.4)	4.28	0.00*	3.06	0.01*
Focal	28 (13.1)	(1.89–5.46)		(1.45–4.80)		10 (4.7)	(1.98–9.23)		(1.29–7.27)	
**Colorectal source**										
Yes	19 (32.3)	2.06	0.02*	1.50	0.28	11(18.6)	2.62	0.01*	2.29	0.09
No	56 (18.7)	(1.11–3.82)		(0.71–3.13)		24 (8)	(1.20–5.70)		(0.87–5.99)	

Abbreviations: BMI stands for body mass index.

*p < 0.05.

Multivariate analysis found that Charlson Grade B (OR = 8.30, 95%CI: 3.58–19.21; p < 0.0001), healthcare-related infections (OR = 6.38, 95%CI: 2.72–14.95; p < 0.0001), sepsis status (OR = 3.98, 95%CI: 1.04–15.21; p < 0.042) and diffuse peritonitis (OR = 3.06, 95%CI: 1.29–7.27; p < 0.011) were independent predictors of all-cause 90-day mortality. (Table 4). The model built predicted 74% of mortality at 90 days in patients presenting with all variables, with Hosmer-Lemeshow goodness-of-fit test significance was 0.48. The model explained 38% (Nagelkerke R2) of the variance and correctly classified 91.1% of cases. The AUROC was 0.887 (95%CI: 0.83–0.93) (Fig. 2B). Post-hoc sensitivity analyses were performed showing confirmatory results. The a-CCI score remained statistically significant at both uni- and multivariate levels when dichotomization using a cutoff of ≤ or > to 1,2,3 and 5 was either performed. Similar results were obtained when excluding the most prevalent and benign disease, complicated acute appendicitis. When analyzed as a continuous scale, the OR for a-CCI was 1.28 (95%CI: 1.14–1.44; p < 0.00) for 90-day postoperative morbidity and 1.60 (95%CI: 1.35–1.90; p < 0.00) for 90-day all-cause mortality (Table 5).

**Table 5:** Post-hoc multivariate sensitivity analyses.

Variables	90-day postoperative morbidity		90-day all-cause postoperative mortality	
	Odds Ratio(95%CI)	P value	Odds Ratio(95%CI)	P value
a-CCI ≤ 1 >	3,29 (1.58–6.84)	0.00	15.45 (2.03–117.84)	0.01
a-CCI ≤ 2 >	2.09 (1.13–3.86)	0.02	9.69 (2.80–33.60)	0.00
a-CCI ≤ 3 >	2.593 (1.43–4.71)	0.00	10.68 (3.84–29.28)	0.00
a-CCI ≤ 5 >	3.99 (2.01–7.95)	0.00	6.34 (2.76–14.58)	0.00
a-CCI excluding acute appendicitis (n = 193)	3.13 (1.55–6.33)	0.00	6.36 (2.62–15.45)	0.00
a-CCI continuous scale	1.28 (1.14–1.44)	0.00	1.60 (1.35–1.90)	0.00

## Discussion

The present study highlights that patient’s degree of comorbidity has a strong and independent impact on the postoperative outcomes following surgery for complicated intra-abdominal infections. Our results shown that the OR of developing severe postoperative complications for patients presenting with a-CCI > 4 is approximately 3.5 times higher than for those who did not. In the same way, and more dramatically, the OR of dying within 90 days after surgery is 8 times greater in patients with a high comorbidity score.

Different tools have been proposed to predict perioperative morbidity and mortality after surgery. Stratifying these risks is crucial to assess the best quality of treatment and can be helpful to compare surgical outcomes between professionals and health-care systems. The American Society of Anesthesiologists (ASA) physical status was developed in 1941 and modified in 1963. It was associated with mortality within 48 h from surgery for patients undergoing both elective and emergent procedures, but noteworthy it is taxed with high inter-observer variability^18^. Other complex scores have been used to stratifying risk of mortality in emergency surgery, such as the Physiological and Operative Severity Score for enUmeration of Mortality and morbidity (POSSUM)^19^, the Emergency Surgery Acuity Score^20^, and the Acute Physiology and Chronic Health Evaluation II (APACHE II) score^21^. There have also been proposed specific scores for assessing the risk of death in cIAI. The World Society of Emergency Surgery (WSES) Sepsis Severity Score is maybe the most reliable and user-friendly of them^4^. It includes among the risk factors the age above 70 years and the immunosuppression status.

The CCI is a simple score, easy to obtain from clinical reports forms and to calculate at the time of admission. It has been commonly used to adjust the outcomes for comorbid conditions^22–24^. Only two studies have addressed its relationship with the adverse outcomes after general emergency surgical procedures, none of them in the setting of cIAI or using a validated classification for postoperative complications^8,25^. Noticeably, we determined the age-adjusted index^7^, as patients’ age has been shown to be an independent predictor of mortality in cIAI^3^. Further, our analyses confirmed the influence of other risk factors for patients presenting cIAI. Early diagnosis and timely therapeutic interventions are crucial steps for improving the treatment outcomes^26^. Moreover, healthcare-related infections are associated with increased mortality and morbidity due to the frequent involvement of at least one multi-drug resistant pathogen and to the poor patient’s health status^27^, in our series 15.4% of the patients presented with healthcare-associated infections similarly to a recent worldwide study (21%)^28^. Diffuse peritonitis^29^ and sepsis status have been previously established as risks factors for mortality in patients with cIAI^4^.

The present findings are limited by the retrospective design of the study. The definition of sepsis changed during the last years, we used the definition of sepsis according to the ACCP/SCCM^13^ published in 1992 as the study period preceded the publication of the Sepsis 3 Consensus^30^. There was few evidence indicating the optimal point for dichotomizing the a-CCI score and we choose the percentile 75^th^, resulting in considering patients with a-CCI more than 4 at the suspected higher risk group. This selection may decrease the power of our findings, but motivated a post-hoc sensitivity analyses confirming the independent impact of a-CCI on the postoperative outcomes.

Although we evaluated a relative large sample size compared to the current literature, the generalization of the results should be done with caution. Howbeit, we analyzed a homogeneous population of patients with cIAI who received emergency surgery, which might be considered the worst-case scenario of intra-abdominal infections at the emergency setting. We thereby focused on the prediction of postoperative adverse complications in a 90-day time frame, which reduces the risk of missing delayed adverse events in particularly vulnerable patients. Moreover, both predictive models presented an adequate fit, and showed an excellent power of discrimination, with AUROC values ranging from 0.815 to 0.887. Other issues related to the use of the a-CCI deserve further commentaries. The index excludes important comorbid conditions that could have a marked influence on postoperative outcomes, as the use of warfarin and non-vitamin K antagonist oral anticoagulants^31^. Also, patient’s history of inflammatory bowel disease, endocrine diseases (i.e., hypopituitarism or adrenal insufficiency), and transplantation are similarly not represented in the score. As they are associated with corticoid and/or immunosuppressive therapies, which have been shown to be clearly related to outcomes in cIAI patients, their influence was not properly evaluated at the present study^4,32^.

Stratifying the risks before surgery in the setting of cIAI is crucial to improve postoperative results and avoid futile treatments. a-CCI score is a simple score which data can be calculated at the time of admission. It allows to assess before the surgery the risk of death and serious postoperative morbidity, helping physicians to made clinical decisions and to optimize treatments or economic resources. Its simplicity it is one of its strengths, being easily reproducible and a useful tool to homogenize treatment groups in future clinical trials.

## Conclusion

The degree of comorbidity, estimated by using an age-adjusted score, showed a critical impact on the postoperative course following emergency surgery for cIAI. Early assessment and management of patient’s comorbidities are mandatory for the decision-making algorithm at the emergency scenario. Although the usefulness of a-CCI is unquestionable, after more than 30 years the development of an updated comorbidity score would be an interesting aim for future multi-centric cohort studies in emergency surgery.

## Data Availability

The data are available on request from the corresponding author.
